# The Effectiveness of FFC-AL-EIT on Psychosocial Outcomes and Care Interactions

**DOI:** 10.1093/geroni/igab046.1324

**Published:** 2021-12-17

**Authors:** Elizabeth Galik

**Affiliations:** University of Maryland School of Nursing, Baltimore, Maryland, United States

## Abstract

This study included a subset of 59 communities and 550 residents from the full FFC-AL-EIT study. Participants were mostly white (98%), female (69%) and had a mean age of 89.30 (SD=7.63). Sites were randomized to the four step FFC-AL-EIT intervention implemented by a function focused care nurse facilitator working with a facility champion over 12 months versus education only. Resident measures included depression, agitation, resistiveness to care and the quality of care interactions and were obtained at baseline, 4 and 12 months. There was a significant positive treatment effect related to depression, agitation, resistiveness to care and quality of care interactions with either less decline or some improvement in these behaviors and symptoms and improvement in the quality of care provided between the treatment versus control group. The study suggests there is some benefit to implementing FFC-AL-EIT for psychosocial outcomes and care interactions among residents in assisted living communities.

